# Post-surgical Euglycemic Diabetic Ketoacidosis in a Patient on Empagliflozin in the Intensive Care Unit

**DOI:** 10.7759/cureus.4496

**Published:** 2019-04-18

**Authors:** Fernand Bteich, Ghassan Daher, Aniruddh Kapoor, Edward Charbek, Ghassan Kamel

**Affiliations:** 1 Internal Medicine, Saint Louis University School of Medicine, St. Louis, USA; 2 Internal Medicine - Critical Care, Saint Louis University School of Medicine, St. Louis, USA

**Keywords:** euglycemic diabetic ketoacidosis, ketoacidosis, empagliflozin, diabetes mellitus, sodium glucose cotransporter

## Abstract

Euglycemic diabetic ketoacidosis (EDKA) is a rare variant of diabetic ketoacidosis which has been recently reported in association with sodium-glucose cotransporter 2 (SGLT-2) inhibitors. Empagliflozin, an agent belonging to this therapeutic class, was approved by the U.S. Food and Drug Administration (FDA) in 2014 for management of type 2 diabetes. Since then, sparse reports of its association with EDKA are emerging, similarly to its predecessors in the class. We report the case of a 58-year-old female who developed EDKA in the intensive care unit (ICU) 48 hours after her last intake of empagliflozin and a day after neurosurgery. Though expected to improve in the post-operative period, she developed a rapidly worsening and unexplained anion gap metabolic acidosis. She was eventually diagnosed with EDKA which was successfully treated with intravenous insulin infusion, dextrose-containing fluids and discontinuation of the offending drug. Metabolic abnormalities improved in less than 24 hours and patient recovered without complications. This report highlights the importance of recognizing EDKA as a complication of oral anti-diabetics and discontinuing SGLT-2 inhibitors days prior to surgery and ICU admission. Care should be applied to providing patient with low-dose ketogenesis-inhibiting basal insulin and close observation of laboratory values in order to minimize delays in diagnosis, prolonged hospital stays and complications of EDKA.

## Introduction

Euglycemic diabetic ketoacidosis (EDKA) is an uncommon acute complication of diabetes mellitus first described by Munro et al. in 1973 [1]. Diagnosis of diabetic ketoacidosis (DKA) is based on laboratory testing showing hyperglycemia (glucose > 250 mmol/L), metabolic acidosis (arterial pH < 7.3 and serum bicarbonate < 18 mEq/L), a high anion gap as well as presence of ketone bodies in the blood or urine of a patient with type 1, or less commonly, type 2 diabetes mellitus [2]. EDKA, unlike classic DKA, is characterized by glycemia <250 mg/dL and typically occurs in the setting of prolonged fasting, persistent vomiting, recent use of insulin, alcoholism and chronic liver disease [2, 3]. Sodium glucose cotransporter 2 (SGLT-2) inhibitors, a relatively new class of oral anti-diabetic agents, have been increasingly associated with incidence of EDKA when a patient is faced with catabolic stress such as surgery or severe illness [4]. This report helps highlight the circumstances during which one should suspect EDKA in a patient, its principles of management and, most importantly, how to prevent its development.

## Case presentation

**Figure 1:**
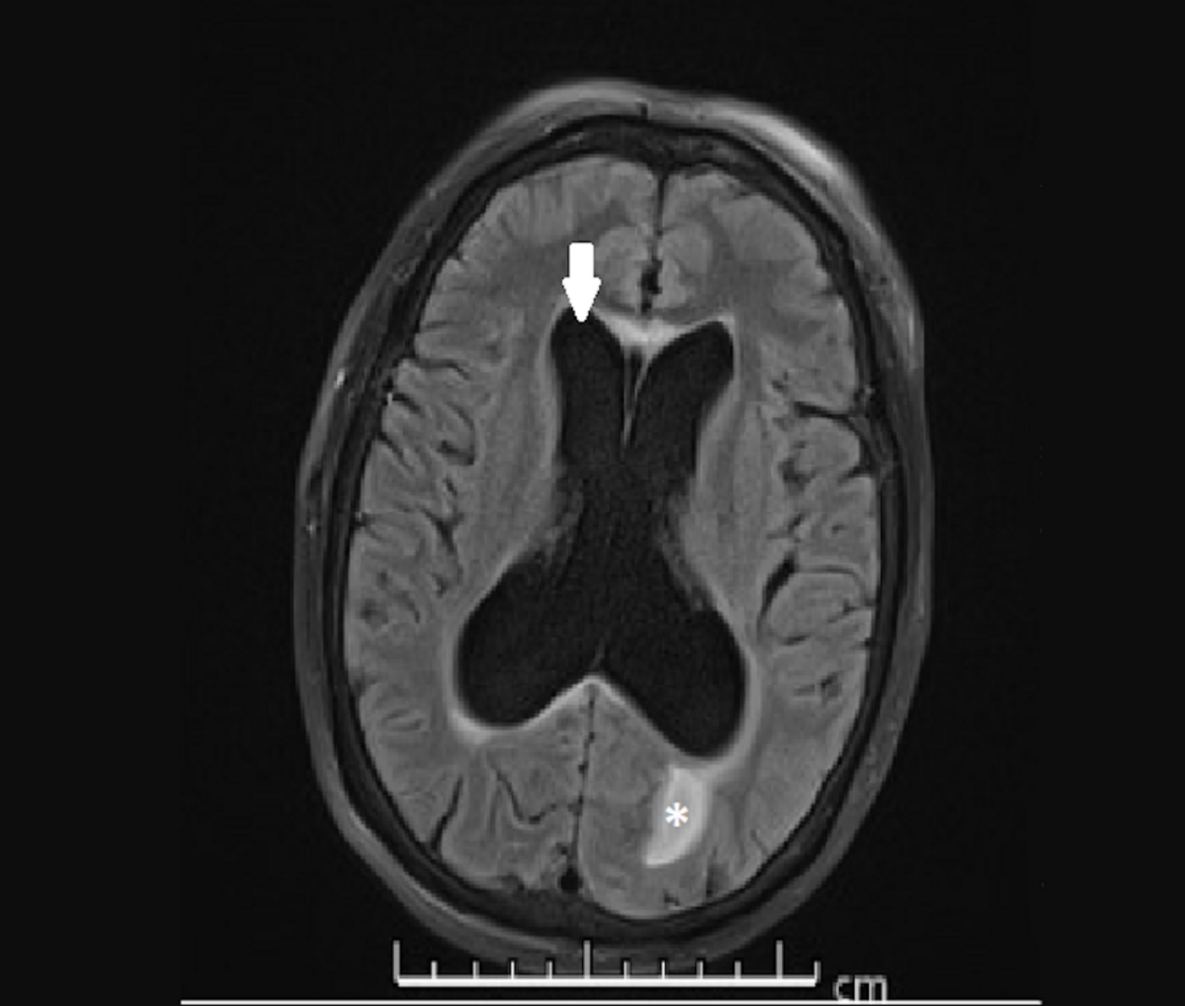
Obstructive hydrocephalus, magnetic resonance imaging (T2 FLAIR sequence). Note enlarged lateral and third ventricles (arrow), with associated transependymal flow of cerebrospinal fluid (asterisk) suggesting acuity of process.

We report the case of a 58-year-old female with history of type 2 diabetes mellitus who was admitted to the medical intensive care unit for altered mental status. Her past medical history was relevant for hydrocephalus requiring ventriculoperitoneal (VP) shunting 25 years ago, essential hypertension and obstructive sleep apnea. The patient was last seen at her baseline mental status three hours prior to presentation. Upon arrival, her primary survey was remarkable for a Glasgow Coma Scale score of 6. No focal neurologic deficits were appreciated. The patient was subsequently intubated for airway protection due to minimal responsiveness. Extensive laboratory workup including complete blood count (CBC), chemistries, urinalysis and illicit drug screen was unrevealing. Magnetic resonance imaging of the brain (Figure 1) showed hydrocephalus involving the lateral and third ventricles with associated trans-ependymal flow of the cerebrospinal fluid (CSF) suggestive of shunt malfunction.

CSF analysis was negative for infection. An electroencephalogram showed non-specific mild right temporal slowing and moderate generalized slowing. A VP shunt exchange was performed on day 2 of the hospitalization after obstruction was confirmed. Nevertheless, the patient’s clinical status worsened and severe metabolic acidosis was noted the following morning (Table 1). Workup was remarkable for a high anion gap (>28 mEq/L), normal lactic acid and elevated serum beta-hydroxybutyrate level (10.09 mmol/L). Arterial pH was 7.20. Blood sugars ranged between 130 and 150 mg/dL. Urinalysis was positive for glycosuria (1000 mg/dL) and abundant ketonuria (>80 mg/dL).

**Table 1:** Laboratory testing during hospital admission. Note the progressive increase in anion gap, decrease in pH and bicarbonatemia with preserved euglycemia. Also note the rapid resolution of diabetic ketoacidosis (DKA) with insulin therapy.

Time after presentation (days)	0	1	2	3	4	5
pH	7.35	7.32	Neurosurgery	7.20	7.37	
Carbon dioxide	24	16		<5	11	21
Anion gap	9	18		>28	16	13
Glycemia (mg/dL)	183	112		143	144	170
Beta-hydroxybutyrate (mmol/L)				10.09	3.58	
Lactic acid (mmol/L)	1.1	1.9		0.7		
Ketonuria (mg/dL)	10			>80		
Glycosuria (mg/dL)	>1000			>1000		

Collateral history obtained from the patient’s family revealed that her diabetes home regimen included insulin glargine 25 units subcutaneously daily, metformin 1000 mg twice daily, glipizide 10 mg daily and empagliflozin 25 mg daily. Her last confirmed intake of oral medications had been on the day prior to presentation. Moreover, it is worth mentioning that the patient was not receiving any enteral nutrition since admission and her diabetes was only being treated with correctional sliding scale insulin while in the intensive care unit. This constellation of information and laboratory findings raised concern for euglycemic diabetic ketoacidosis in the setting of SGLT-2 inhibitor use. The patient was therefore treated with an intravenous insulin infusion. Dextrose-containing maintenance fluids were added to avoid hypoglycemia. Ketoacidosis resolved over 48 hours (see evolution in Table 1). The patient was eventually transitioned to subcutaneous basal-bolus insulin regimen and started on tube feeds. No relapse of her EDKA occurred for the remainder of her hospital stay.

## Discussion

This case illustrates the well-known role of surgery in triggering ketoacidosis. Counter-regulatory hormones, namely, cortisol, glucagon, growth hormone and catecholamines, are secreted at high levels in the post-operative setting [5]. These hormones help create a milieu of insulin resistance which releases the brakes off lipolysis. Degradation of adipose tissue triglycerides leads to a massive efflux of free fatty acids (FFAs) to the liver which is the main site of conversion of FFAs to ketones. On the other hand, in classic DKA, patients also develop hyperglycemia due to accelerated glycogenolysis and an increase in gluconeogenesis [3]. Despite insulin resistance and relative insulin deficiency, patients on SGLT-2 inhibitors are generally normoglycemic or moderately hyperglycemic. This phenomenon is likely related to constant glycuresis achieved by co-transporter inhibition in the kidneys or decreased glucose production by the liver during fasting state. Continuing home insulin, even at lower doses, can also help keep glycemia in check. Furthermore, loss of glucose, and therefore calories, in urine is thought to create a certain degree of “starvation” which stimulates glucagon secretion, suppresses endogenous insulin and results in further production of ketones [6].

Our patient’s intake of empagliflozin one day before hospitalization, persistence of its effect manifesting as glucosuria for more than 48 hours post-interruption, concurrent increase in stress hormones after surgery and absence of adequate calorie intake all resulted in a decrease in the insulin:glucagon ratio and post-operative genesis of EDKA.

With recent large studies confirming cardio and reno-protective properties of SGLT-2 inhibitors [7, 8], and with the new American Diabetes Association (ADA) guidelines [9] and the 2018 American College of Cardiology (ACC) Expert Consensus Decision Pathway [10] recommending consideration of gliflozins as add-on to metformin in a large proportion of diabetics with cardiovascular disease, it is expected that SGLT-2 use will only increase over time. Physicians including internists, intensivists and emergency physicians should all be aware of EDKA as a potential complication of diabetes therapy, especially in contexts of emergent surgery or discontinuation of insulin. Careful monitoring of daily laboratory values for development of gap acidosis is warranted during the first few days of hospitalization even if the oral SGLT-2 inhibitor has been stopped prior to admission for its effects can sometimes last more than a week [11]. On the other hand, starting patients on a prophylactic low dose of long-acting insulin after cessation of oral agents once in hospital seems like a reasonable strategy to mitigate the risk of developing EDKA in at-risk populations. Prevention remains essential: educating patients taking SGLT-2 inhibitors to stop the agent 48 hours prior to surgery, prompt reporting of persistent gastro-intestinal symptoms (nausea, vomiting, abdominal pain) to healthcare provider and use of urine ketone strips to screen for ketonuria are all effective measures which can help reduce the occurrence and morbidity of EDKA.

## Conclusions

SGLT-2 inhibitors like empagliflozin are known to increase the risk of euglycemic diabetic ketoacidosis, a condition some physicians may be unfamiliar with. This report adds to the growing body of literature demonstrating the risks encountered with surgery, prolonged starvation and insulin cessation in patients treated with these agents. Education of both physicians and patients is crucial to avert or decrease the negative impact of this complication.
